# Isolated Menarche and Empty Sella Turca: A Rare Pediatric Case

**DOI:** 10.7759/cureus.100275

**Published:** 2025-12-28

**Authors:** Mariana Vieira, Inês Alexandra Azevedo, Maria Adriana Rangel, Rosa A Campos, Ana L Leite

**Affiliations:** 1 Pediatrics, Unidade Local de Saúde Viseu Dão-Lafões, Viseu, PRT; 2 Pediatrics, Unidade Local de Saúde Entre Douro e Vouga, Santa Maria da Feira, PRT; 3 Pediatric Endocrinology, Unidade Local de Saúde Gaia/Espinho, Vila Nova de Gaia, PRT

**Keywords:** adrenal insufficiency (ai), empty sella turcica, paediatric endocrinology, precocious puberty (pp), short stature (ss)

## Abstract

Isolated menarche is a rare condition characterized by vaginal bleeding without other pubertal signs. Primary empty sella is usually an incidental finding but may be associated with pituitary dysfunction. We report a 10-year-old girl evaluated for short stature and two episodes of vaginal bleeding. She showed a prepubertal stage, delayed bone age, and a prepubertal uterus and ovaries on ultrasound. Magnetic resonance imaging revealed a primary empty sella. Progressive growth failure and an inadequate response to a stimulation test led to the initiation of growth hormone therapy at 13 years. At 14 years, she developed symptoms of adrenal insufficiency supported by a low morning cortisol value, requiring hydrocortisone treatment. Puberty progressed spontaneously, and menarche occurred at 15 years. At 17 years, she continues treatment with improved growth and resolution of symptoms.

## Introduction

Isolated menarche is a rare condition with uncertain etiology, characterized by the onset of an isolated or recurrent vaginal bleeding without secondary sexual characteristics [1,2]. Primary empty sella is often discovered incidentally. In this rare condition, the sella turcica is typically filled with cerebrospinal fluid, leading to compression of the pituitary gland, which can cause endocrine dysfunction [3]. Early recognition and treatment of hormonal deficiencies are critical to improving outcomes.

## Case presentation

We present the case of a 10-year-old female referred to the pediatric endocrinology clinic for short stature and suspected of premature menarche. Her parents reported a one-year growth arrest. Additionally, two episodes of vaginal bleeding were described, occurring eight and five months before the evaluation, with the first episode lasting five days.

The patient complained of intermittent mild headaches. She denied symptoms such as leucorrhea, vulvovaginitis, urinary tract infections, abdominal pain, alopecia, bowel dysfunction, polydipsia, vision changes, or other symptoms. No significant medical history or regular medications were reported.

She was born at 40 weeks following an uncomplicated pregnancy. Her birth weight was 3.36 kg (-0.28 standard deviation score, SDS), length 47 cm (-1.15 SDS), and head circumference 34.5 cm (0.52 SDS). There were no neonatal complications.

Parental pubertal development was normal. Her father's height was 174 cm, and her mother's was 162 cm. The patient's target height was 164.1 cm (25-50th percentile of WHO growth charts, -0.19 SDS). No relevant family medical history was identified.

At the age of nine, her height was 126 cm (3rd-15th percentile, -1.37 SDS). Growth deceleration with gradual crossing of percentiles was observed afterwards, until it became below the 3rd percentile.

At 10 years and six months, her height was 128 cm (<3rd percentile, -2.24 SDS), her weight was 23.3 kg (<3rd percentile, -2.20 SDS), and her body mass index was 14.2 kg/m^2^ (3rd-15th percentile, -1.62 SDS). The examination revealed a nondysmorphic, proportionate patient who seemed younger than her chronological age. Sexual development was Tanner stage 1. No other abnormalities were noted.

Laboratory results (Table 1) revealed normal Insulin-like growth factor 1 (IGF-1) levels (125 ng/mL) and prepubertal levels of sex steroids and gonadotropins. Chromosome analysis demonstrated a 46,XX karyotype. Her bone age was eight years and ten months (20 months delay). 

**Table 1:** Laboratory results at 10 years and six months ACTH - adrenocorticotropic hormone; DHEA-S - dehydroepiandrosterone sulfate; FSH - follicle-stimulating hormone; IGF-1 - insulin-like growth factor 1; LH - luteinizing hormone; TSH - thyroid stimulating hormone; 17-OH-P - 17-hydroxyprogesterone

Laboratory results	Patient's value	Normal range
FSH (mUI/mL)	0.64	0.06-3.4
LH (mUI/mL)	<0.10	<0.3
Estradiol (pg/mL)	<5.00	12.3±11.9
TSH (uUI/mL)	2.23	0.27-4.20
Free T4 (ng/dL)	1.34	0.93-1.70
Prolactin (ng/mL)	11.80	4.79-23.30
DHEA-S (ug/dL)	12.00	40-130
Delta-4-androstenedione (ng/mL)	0.30	0.4-17.5
17-OH-P (ng/mL)	0.40	<2
ACTH (pg/mL)	14.50	0-46
Cortisol (ug/mL)	15.70	6.2-19.4
IGF-1 (ng/mL)	125	111-551

**Figure 1:**
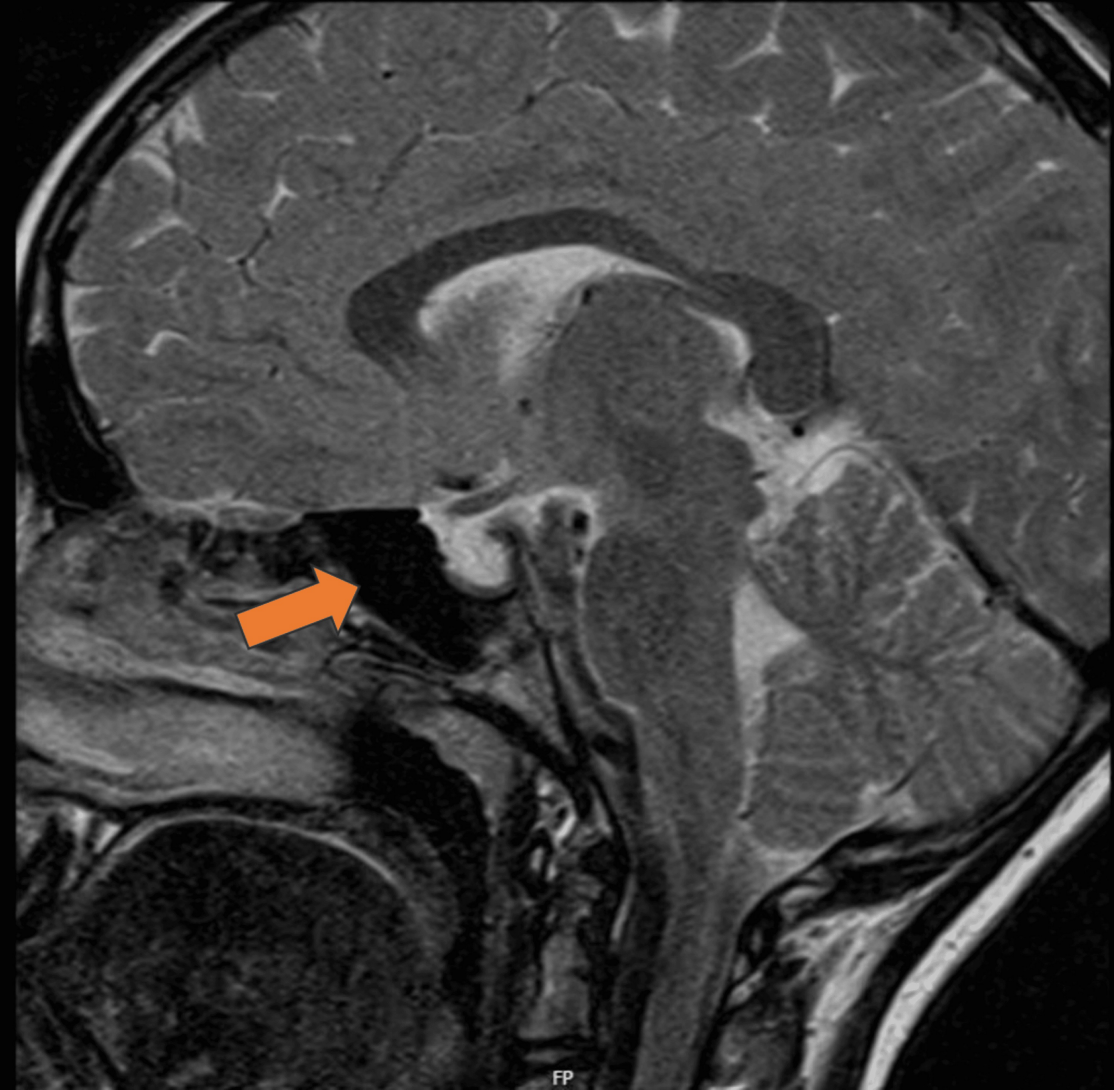
Empty sella turca on MRI

The pelvic ultrasound revealed a prepubertal uterus and ovaries. The uterus measured 0.8x1.4x2 cm (anteroposterior, transverse, and length, respectively), and the ovarian volumes were 3.3 cc on the right and 2.4 cc on the left. Cranial magnetic resonance imaging described a classic pattern of empty sella turca (Figure 1). 

Growth hormone (GH) stimulation testing with clonidine revealed peak GH levels of 8.36 ng/mL at 90 minutes. She was followed up regularly, and at 13 years and one month, significant growth deceleration (137.6 cm; <3rd percentile, -2.76 SDS) prompted initiation of subcutaneous somatropin therapy (0.035 mg/kg/day).

At 14 years and one month, she complained of low tolerance to fasting with sporadic nausea and vomiting. At that time, her laboratory results showed a low basal cortisol level (8 AM, 5.1 ug/dL), adrenocorticotropic hormone (ACTH) level in the lower half of the reference range (9.1 pg/mL), and a normal ACTH stimulation test. A diagnosis of central adrenal insufficiency was made, and treatment with hydrocortisone started (8 mg/m^2^/day).

Puberty progressed gradually, with thelarche at 13 years, pubarche at 14 years, and menarche at 15 years. Currently, at 17 years, she maintains regular follow-up and therapy with somatropin (0.037 mg/kg/day) and hydrocortisone (11 mg/m^2^/day). There has been an improvement in growth with her height being 155.6 cm (10-25th percentile, -1.09 SDS). Moreover, symptoms of adrenal insufficiency have resolved.

## Discussion

Isolated menarche is a rare condition, requiring additional investigation (e.g., hormonal profile, bone X-ray, pelvic ultrasound) for adequate management and follow-up. It is a diagnosis of exclusion, with the main differential diagnoses being ovarian cysts, infections, trauma, malignancy, foreign bodies, precocious puberty, estrogen exposure, or McCune Albright syndrome [1,2,4]. When facing a case of isolated menarche, there are typically no signs of advanced skeletal maturation, and the adult height is normal. Additionally, fertility is generally not affected [1,2].

Primary empty sella is often discovered incidentally and can cause endocrine dysfunction [3]. The most common endocrine disturbances involve the somatotropic and gonadotropic axes, with dysfunction of multiple pituitary axes occurring more frequently than isolated axis dysfunction [3,5,6]. Therefore, hormonal testing should be performed in all diagnosed cases.

The diagnoses of isolated menarche and empty sella are not related. However, in this case, the suspicion of the first one enabled the diagnosis of the second.

## Conclusions

This case highlights the value of correlating radiologic findings with clinical symptoms to guide appropriate intervention. Ongoing monitoring allows clinicians to detect subtle changes in pituitary function that may progress over time. Ensuring timely evaluation and multidisciplinary collaboration can help optimize long-term management and reduce the risk of complications associated with pituitary dysfunction.
